# Simultaneous optimisation of earwig hindwings for flight and folding

**DOI:** 10.1242/bio.016527

**Published:** 2016-04-25

**Authors:** Julia Deiters, Wojciech Kowalczyk, Tobias Seidl

**Affiliations:** 1 Westphalian Institute for Biomimetics, Westphalian University of Applied Science, Muensterstr. 265, Bocholt 43697, Germany; 2 Department of Mechanics and Robotics, University of Duisburg-Essen, Lotharstr. 1, Duisburg 47057, Germany

**Keywords:** Earwig hindwing, Structural stabilisation, Wing folding, Insect flight, Passive wing control, Dermaptera

## Abstract

Earwig wings are highly foldable structures that lack internal muscles. The behaviour and shape changes of the wings during flight are yet unknown. We assume that they meet a great structural challenge to control the occurring deformations and prevent the wing from collapsing. At the folding structures especially, the wing could easily yield to the pressure. Detailed microscopy studies reveal adaptions in the structure and material which are not relevant for folding purposes. The wing is parted into two structurally different areas with, for example, a different trend or stiffness of the wing veins. The storage of stiff or more flexible material shows critical areas which undergo great changes or stress during flight. We verified this with high-speed video recordings. These reveal the extent of the occurring deformations and their locations, and support our assumptions. The video recordings reveal a dynamical change of a concave flexion line. In the static unfolded state, this flexion line blocks a folding line, so that the wing stays unfolded. However, during flight it extends and blocks a second critical folding line and prevents the wing from collapsing. With these results, more insight in passive wing control, especially within high foldable structures, is gained.

## Abstract (precis)

**Summary:** Adjustments of the wing structure in earwig hindwings enable passive wing control during flapping flight. With an expansion of the claval flexion line, the highly foldable wing can withstand deformations during flapping flight and stay unfolded.

## INTRODUCTION

Insect wings and their broad variety have triggered research in many ways. Subjects of interest include material properties of substructures within the wings (Hepburn and Chandler, 1980; Lomakin et al., 2011), structural arrangement of the veins (Wootton, 1992; Combes and Daniel, 2003a), folding purposes (Saito et al., 2014; Geisler, 2012; F. Haas, MPhil thesis, University of Exeter, 1994; F. Haas, PhD thesis, Friedrich Schiller University Jena, 1998) or analysis of the hovering or flapping flight (Sane, 2003; Lehmann et al., 2011; Vogel, 1966; Dickinson et al., 1999; Fry et al., 2005). Unravelling the interaction of these various aspects is of great importance for understanding the performance of the wings during flight and on ground. Folding structures, for example, are a result of both material properties and wing structure arrangements (Haas et al., 2000b; Wootton, 1992).

Insects employ different folding methods to protect and maintain the functionality of their wings on ground (Saito et al., 2014; Muhammad et al., 2009; F. Haas, MPhil thesis, University of Exeter, 1994; F. Haas, PhD thesis, Friedrich Schiller University Jena, 1998). Without a proper protection, the wings – especially very thin wings – could be easily damaged. Social wasps (Vespidae), for example, turn their wings sideward and fold the forewings with one longitudinal fold (Danforth and Michener, 1988). Beetles (Coleoptera) protect their membraneous hindwings by folding them with longitudinal and/or transversal folds (Saito et al., 2014; F. Haas, MPhil thesis, University of Exeter, 1994; F. Haas, PhD thesis, Friedrich Schiller University Jena, 1998) and storing them under the sclerotised forewings – called Elytra. In earwigs (Dermaptera) we find a unique and highly complex mechanism: the hindwings are folded by a combination of three techniques (transversal, longitudinal and fan-like folding) and subsequently stored under the reduced forewings (Haas and Wootton, 1996; Kleinow, 1966; F. Haas, MPhil thesis, University of Exeter, 1994).

**Fig. 1.**
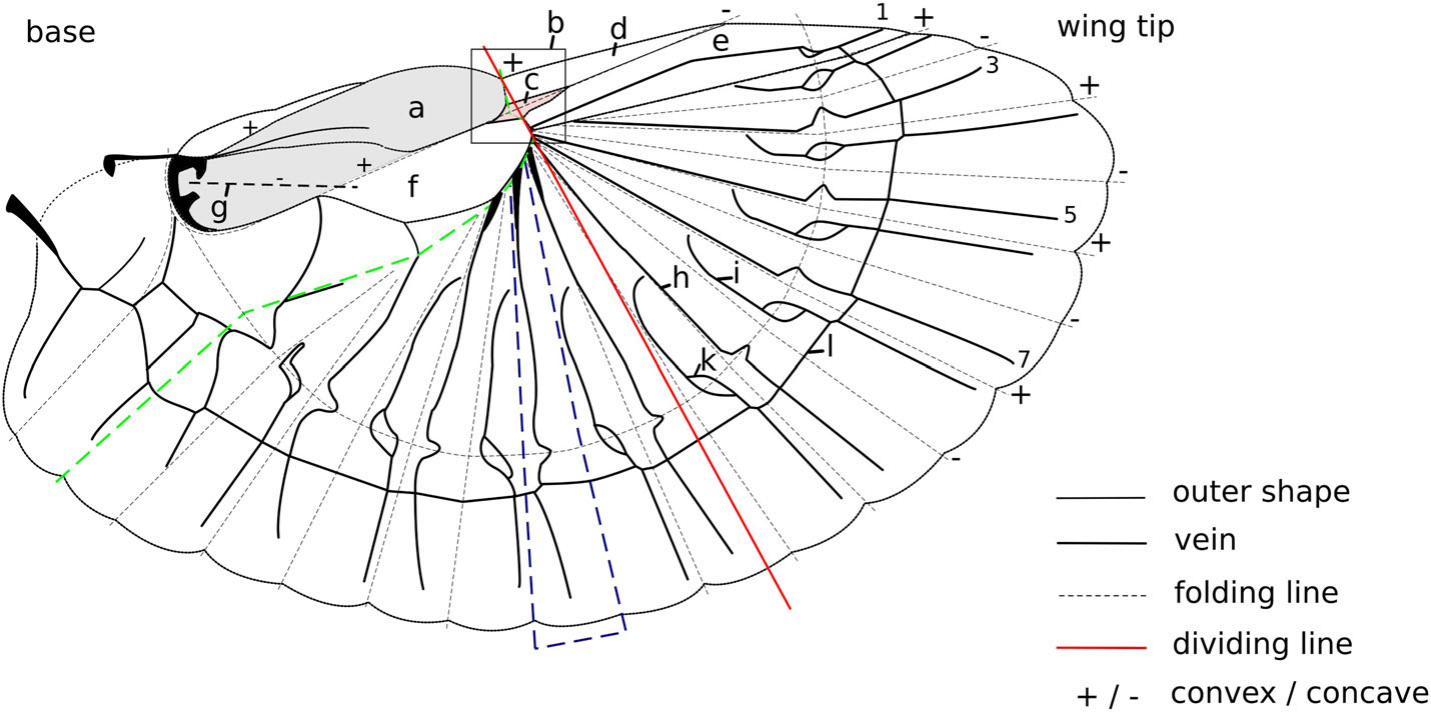
**Schematic wing of *Labia minor*.** A, squama; B, mid-wing mechanism; C, central area; D, outer apical area; E, inner apical area; F, ulnar area; G, claval flexion line; H, radial vein; I, intercalary vein; K, patch (broadened area); L, ring cross vein; the numbers represent the vein number. Shaded in grey is the surface area of the folded state. Green dashed line highlights the folding line running through the central area, between ulnar area and fan and ends near the wing base at the end of the fan. Blue dashed line shows the shape of a single fan unit.

Most work about earwig hindwings considers the folding process or the stability in the unfolded but static state (Kleinow, 1966, 1970; Haas et al., 2000b) and highlighted their optimised folding ability. Two important mechanisms were observed by Kleinow (1966) and Haas (F. Haas, MPhil thesis, University of Exeter, 1994): (i) the mid-wing mechanism, situated in the central area of the wing and (ii) the claval flexion line (Fig. 1B,G). Both of these mechanisms require a ‘snap in’ to lock the deployed wing and prevent it from collapsing under load (Kleinow, 1966; F. Haas, MPhil thesis, University of Exeter, 1994). After release of the ‘snap in’, the wing folds back with a reported folding ratio of 1:10 for *Forficula auricularia* (Haas and Wootton, 1996). Our own data even show folding ratios reaching 1:18 in *Labia minor* (Deiters et al., 2015). The mechanical challenge of achieving this high packing ratio is only surpassed by managing a reliable folding process and maintaining a stable deployed wing during flapping flight.

Contrary to common belief, some earwig species are quite avid fliers (Fritze, 1916; Kleine, 1921; Meineke, 1990; Buzzetti et al., 2003). Kleinow (1966) observed a flying speed of 0.2 to 0.5 ms^−1^ for *Labia minor* during flapping flight. From his observed data we can conclude that earwigs belong to the so-called ‘slow-flying’ insects. According to Wootton (1992) the angular velocity of flapping in slow-flying insects is far greater than the forward velocity and the wing tips move faster than the wing base. In his work he also mentioned that insects with extensively twisting wings and a high structural flexibility are often associated with the ability to fly slowly and sometimes to operate at a wide range of speeds with a high degree of manoeuvrability. By contrast, fast flight is mostly restricted to insects with rigid non-twisting wings.

During flapping movements the wing supinates and pronates. This leads to different wing configurations towards the direction of flight and requires the adaptation of the wing to the new position. A slightly cambered wing helps to generate lift (Vogel, 1967). To obtain optimal lift during up- and downstroke, the wing needs to reverse the camber after supination and pronation. For this a near-optimal balance between stiff and flexible material is needed (Anderson, 2012).

In insect wings, active muscles are only found at the wing attachment to the insect body (Wootton, 1992), yet the wing can deform considerably (Ennos, 1988). This deformation is controlled passively through the architecture and the interaction of the different parts of the wing: leading-edge, vein structure, material distribution and properties as well as flexion lines which lead to the important spanwise stiffness and chordwise flexibility (Wootton, 1981, 1992; Combes and Daniel, 2003b). The highly elastic protein resilin plays a vital role in the control of deformation and elasticity which supports folding processes and bending of the veins (Haas et al., 2000a,b).

The flapping flight of earwigs (around 50 Hz; Kleinow, 1966) necessitates a transition between the stable folded state of the wings and the stable unfolded state, which is a major challenge. If our assumption is correct and earwigs belong to slow flying insects, we expect that (i) their wings are very flexible and exhibit significant deformations during flight, (ii) their wings reveal extensive distribution of resilin in the veins to support those deformations and (iii) clearly identifiable flexion lines adjust the wing's shape during flight. Then of course the question arises how the two mechanisms (mid-wing mechanism and claval flexion line) stay in place during wing deformations, for example during camber reversion.

We examined these hypotheses by first mapping the wing geometry to high detail, determining stiffening and elastic elements, and verified the findings by means of using free flight high-speed cinematographic observations.

## RESULTS AND CONCLUSIONS

### Wing structure and material distribution

**Fig. 2.**
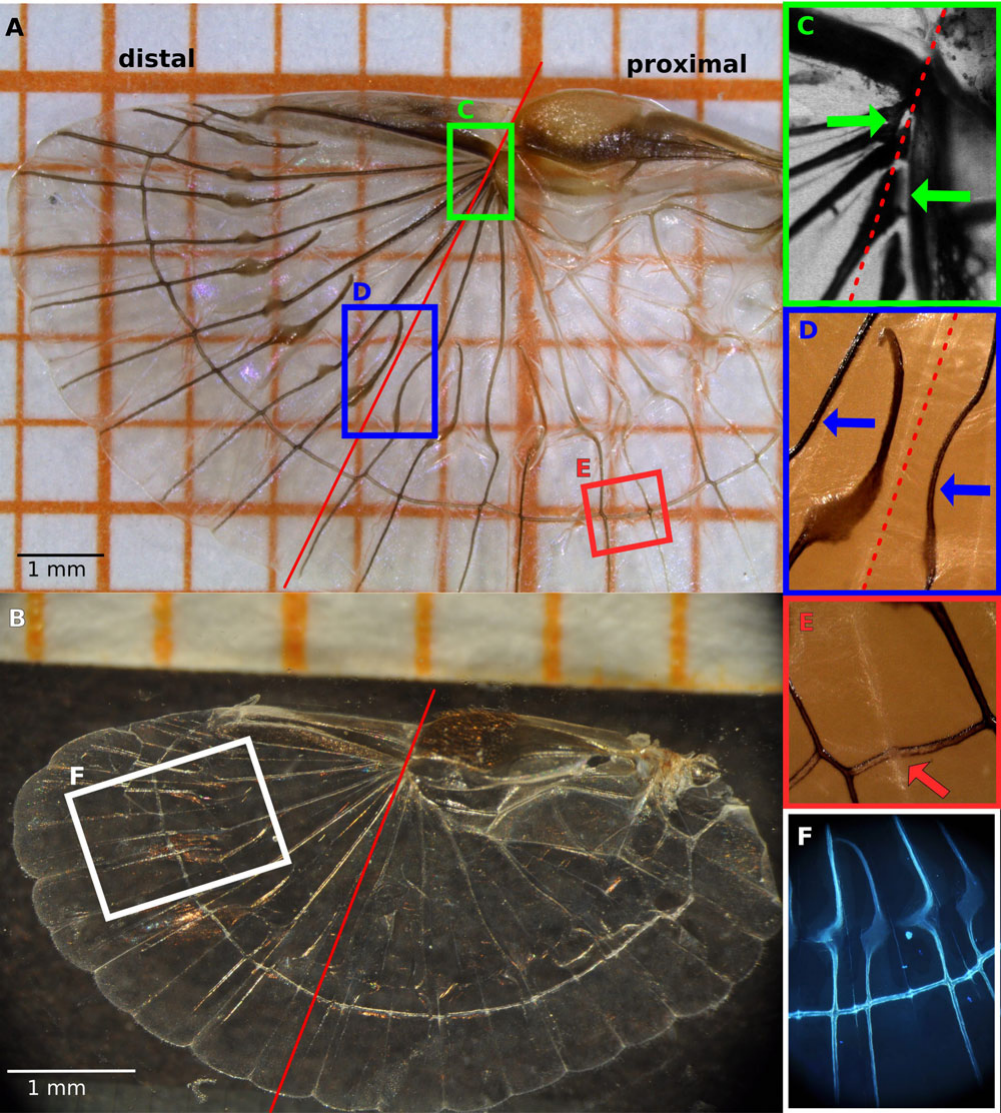
**Unfolded dermapteran wings.** (A) *F. auricularia.* (B) *L. minor.* The vertical dividing line (red) indicates the functional division of the wing. (C) In the distal part (left), the radial veins are joined, while in the proximal part they are separate and have a broadened base (green arrow). (D) Radial veins are either straight or curved (blue arrows). (E) Along the folding line, the crossing vein exhibits a reduced degree of sclerotisation (orange arrow). (F) The veins of *L. minor* show an overall distribution of resilin.

Strong tanning can be found in the leading edge of the wings of *F. auricularia*. The edge consists of three areas, namely the inner and outer apical areas and the squama (Figs 1 and 2). Judged by the intensity of the cuticle tanning (Chapman, 1982), the leading edge can be assumed to be very stiff. Only the central area (Fig. 1C) is almost transparent and hence more elastic (Fig. 2A,B). Next (beneath) the squama lies the ulnar area with differently tanned regions: The region adjacent to the apical area and vein origin as well as the region around the claval flexion line are stained brown, the rest is more transparent (Fig. 2A,C).

In the longitudinal wing veins, tanning – and hence stiffness – decreases from a strong staining in the wing tip (*F. auricularia*) to a lesser one near the base (Fig. 2A), which indicates a stiff outer and a flexible inner wing. The tanning in the radial veins of the more proximal part changes within the veins: It is stronger at the origin (next to the ulnar area), then decreases until after the patch where it intensifies at first and then decreases again after crossing the ring cross vein (Fig. 2A). The tanning in the intercalary veins of this region is similar, except that these are nearly transparent at the beginning and show the pattern of the radial veins after the patch only. The ring cross vein is less tanned on the crossings of the folding lines (Fig. 2E). *L. minor* shows a similar pattern, but with a lighter tanning and almost fully transparent veins (Fig. 2B). Contrary to *F. auricularia*, the tanning of the longitudinal distal veins decreases obviously in direction to the wing's trailing edge.

The trailing edge of the wing has no supporting edgings. The only stiffened structures within the trailing edge are the tapered ends of the wing veins. Compared to the leading edge, the trailing edge is flattened out similar to an aerodynamic profile.

The reduced sclerotisation and hence increased transparency of the veins in the wings of *L. minor* help to identify resilin as already mentioned in Deiters et al. (2015). Because of lesser sclerotisation and therefore reduced stiffness of the veins containing the elastic protein resilin, these veins exhibit an increased elasticity. This is not only important for wing folding, but also for flight — particularly for flapping flight, where the increased elasticity permits adjusting the wing shape (see ‘Free flight analysis’). Microscopy analyses revealed an intensive distribution of resilin in the complete vein system (Fig. 2D) and additionally in the central area as Haas et al. (2000b) also found for *F. auricularia*.

### Vein arrangement

In both species, different vein structures and geometries within the wing lead to a structural division (Fig. 1; Fig. 2A,B, red line) (Deiters et al., 2015). At this line, the wing is divided into a structurally different stiff outer (distal) and a more compliant inner (proximal) region. The most significant characteristics leading to this partitioning are listed in Table 1 and illustrated in Fig. 2.

**Table 1.**
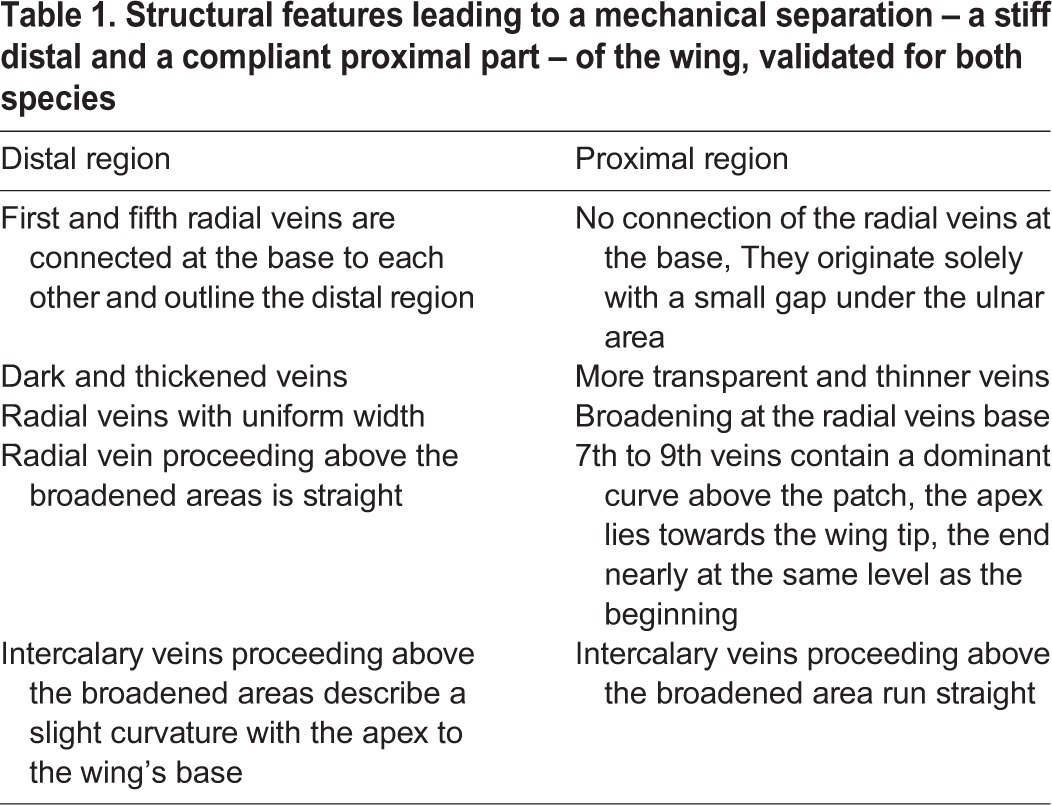
**Structural features leading to a mechanical separation – a stiff distal and a compliant proximal part – of the wing, validated for both species**

In *F. auricularia* the radial veins patch is integrated in the above mentioned curvature of the proximal part (Table 1). The curvature terminates together with the patch. In *L. minor* the patch starts after the curvature. The curvature increases within the proximal part from the wing base to the vertical dividing line. The vein curvature seems to be unimportant for the folding process. Perhaps it has a beneficial effect during flight and the occurring wing shape adjustments.

Through the folding lines (dashed lines in Fig. 1) the fan is separated into several units. These units are shaped as an acute-angled triangular region where the pointed tip is situated next to or underneath the ulnar area. Stabilising the surrounding area, a longitudinal (radial) vein emerges near the base of the acute angle, proceeding along the inner folding line until nearly half of the vein length and then changing its course towards the outer folding line. The change in direction often takes place within the patch or during crossing the ring cross vein (Fig. 1, Fig. 2A). Longitudinal intercalary veins initiate at the outer folding line, describing a curve to the inner folding line and then follow the same proceeding. All longitudinal veins are inter-connected by a ring cross vein. This vein stabilises the wing structure and leads to interdependent movements of the veins and therefore facilitates the (un)folding process.

As pointed out earlier (Deiters et al., 2015), the observed partition of the wing emerges from managing changing loads during flapping flight. The partition (Fig. 1, red line) is not a distinct structure. Its location can be deduced from the resulting mechanical behaviour only, which is the result of structural adaption dependent on the appearing loads during flight and the position of the influenced structure (see ‘Free flight analysis’).

**Fig. 3.**
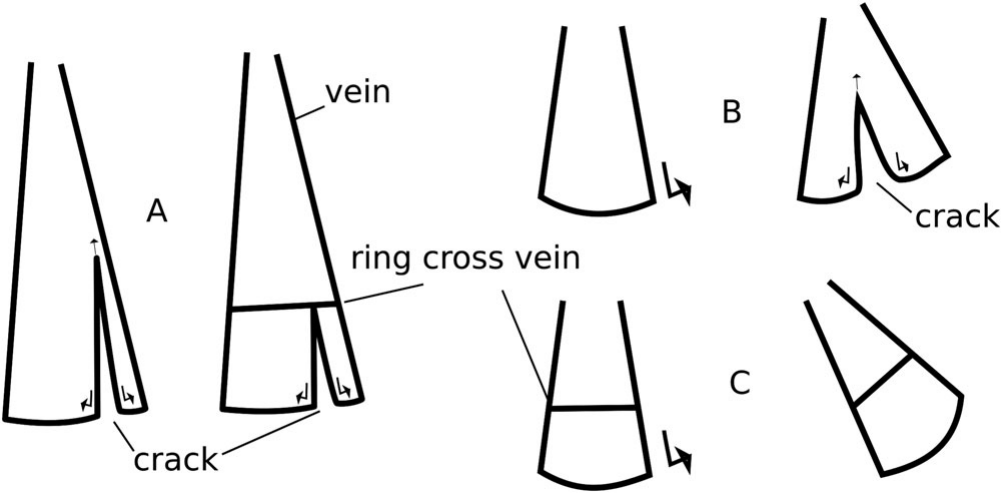
**Mechanical features of the wing cross vein.** (A) Stop of rip propagation (right) versus a hypothetical situation without the vein (left). (B) Ripping event resulting during folding. Lateral forces put high strain on the membrane while (C) at the presence of a ring cross vein, the tearing forces are transmitted through the vein.

The specific triangular arrangement of the radial and intercalary veins supports the stretching of the membrane in air and also the stabilisation of the fan units during the folding process. Through the ring cross vein, the longitudinal veins are kept at distance and the membrane is prevented from wrinkling up (Ennos, 1988). Additionally the ring cross vein prevents the longitudinal veins from drifting apart, for example during the folding process, and protects the membrane from ripping caused by too much initial stress (Fig. 3A,B). In case the membrane is already ripped, the ring cross vein serves as a mechanical barrier stopping the propagation of the tear and maintains the functionality of the wing (Fig. 3A) (Wootton, 1992).

### Free flight analysis

The structural differences leading to a separation of the wing into a structurally more rigid distal and a less rigid proximal part were furthermore scrutinised by high-speed video analysis.

**Fig. 4.**
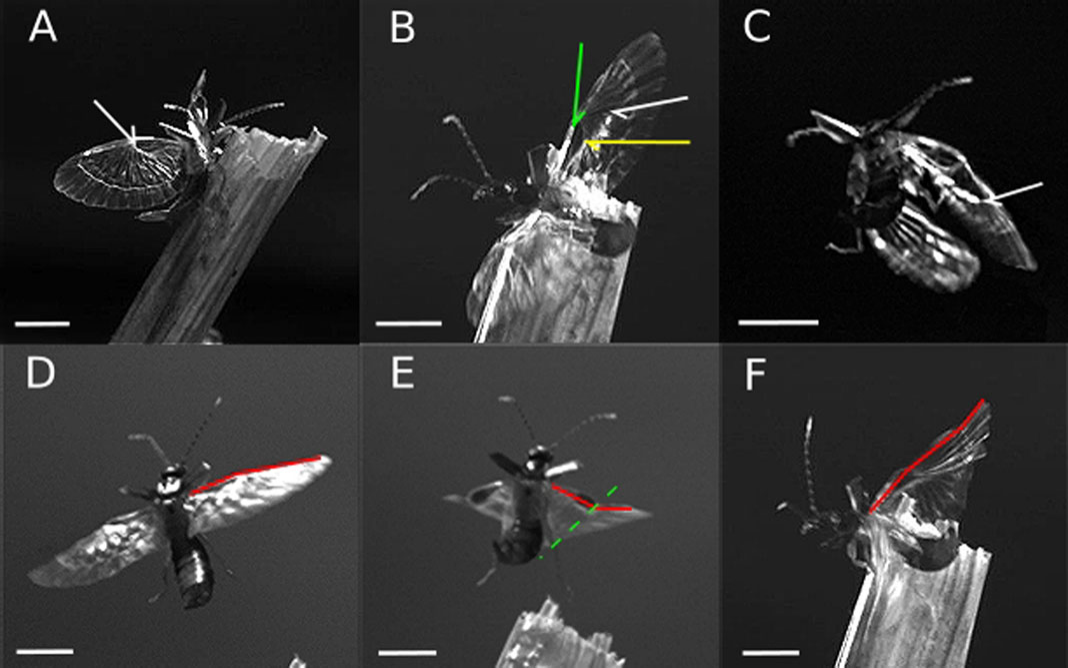
**Folding inhibition and wing deformation of *L. minor***; (A-C) folding-inhibition through the claval flexion line. (A) Resting position, the claval flexion line inhibits only the folding line within the ulnar area. The tip of the white arrow indicates the end of the resulting deformation. (B) Downstroke during take-off, squama is slightly tilted forward (green arrow indicates the flexion line behind the squama), pronation of the wing is initiated, the yellow arrow indicates the end of the claval flexion line, the white arrow the end of the deformation. (C) Deformation of the wing during upstroke within a curve flight, white arrow indicates end of the deformation caused by the inhibition through the claval flexion line. (D-F) Shape-changes of the leading edge (red line) during flight. (D) Downstroke, the leading edge of the wing shows a slight kink. (E) Deformation during upward movement, the leading edge is more pointed out, the deformation transition within the wing is nearly situated at the vertical dividing line (green dashed line). (F) Deformation during downstroke, the trailing edge is moving along with a short delay starting at the ring cross vein. All images are sharpened using Inkscape for a clearer texture. Scale bar: 2 mm. Images taken at 1000 fps, 201 μs shutterspeed.

During flight, only the squama is actively driven by muscles and often slightly tilted forward (Fig. 4B). The remaining wing sections are mechanically coupled and hence change their shape accordingly to the squama movement and the surrounding windage. The leading edge consists of squama and outer apical area where the central area connects both elements and acts as an elastic joint (Fig. 1). It enables the outer wing to move differently from the squama movement.

More so, the central area is located between squama, outer apical area, inner apical area and ulnar area and contains the mid-wing mechanism (Kleinow, 1966). In the deployed state, these regions form a pyramidal shape (F. Haas, MPhil thesis, University of Exeter, 1994). This shape leads to a kink in the leading edge which supports a slightly reversed V-like cambered profile (Fig. 4D,E, the red line outline the leading edge). The resulting angle varies throughout the wing-beat cycle: during the downward movement the angle is more flattened out (Fig. 4D,F) and more pointed during the upward movement (Fig. 4E). A folding line which also acts as a flexion line (Fig. 1, green dashed line) helps to enable this movement.

According to the angular change, the distal wing part complies with the loading pressure of the air. It bends upwards during down-stroke and downwards during the upward stroke. This movement is carried out through the joined veins in the distal part and the resulting wing-bending ends nearly at the vertical dividing line (Fig. 4E).

The proximal part behaves almost independently from the kink. Here the claval flexion line stabilises the wing. During rest, the claval flexion line is only slightly buckled against the formed camber of the wing (Fig. 4A). While the claval flexion line prevents the convex folding line within the ulnar area from folding and bending, it ensures the stability of the proximal part. During upward movement of the wing, the wind load bends the distal part downwards, and hence the buckling of the claval flexion line increases (Fig. 4C). In addition, the buckling pushes in the convex folding line between ulnar area and fan (Fig. 1, green dashed line) at which the distal part is moving up and down. As a result this prevents an initiation of the folding process caused through to strong deformations. Through this buckling, two important folding lines are blocked, and hence the wing is locked in its deployed position. It does not fold away under the squama and lose its function.

The curved veins in the proximal part and also the changing tanning seem to be a consequence of the buckling. At the end of the buckled wing portion, in the direction of the trailing edge, the wing bulges out again (Fig. 4B,C). This region appears to coincide with the region of lesser sclerotisation and the strong vein curvature in the proximal part.

Since the proximal part does not bend uniformly and heavily as the distal part, we assume that the veins do not need to withstand similarly strong forces and therefore can be less sclerotised. Furthermore the veins in the proximal part need to be more flexible as in the distal part for the deformations, so less sclerotisation would be advantageous.

The area between ring fold and trailing edge often follows the wing deformation with a short delay and therefore supports a slightly cambered profile (Fig. 4F). Together with the V-like arrangement of the leading edge, the wing forms a continuous slightly cambered profile (Fig. 4C-F).

Wootton (1992) indicates that the functions of folding and flexion lines can be combined. Here such a combined folding line within the central area also serves as a flexion line during flight and hence supports the idea that the properties of folding and flying can act together and support each other. This line originates within the central area where it helps bending down the wing tip. In addition, the folding line directly behind the squama also acts as a flexion line at which the squama is slightly tilted forward.

## DISCUSSION

So far the structure of earwig wings was analysed with regards to the static unfolded state of the wings (Kleinow, 1966; Haas et al., 2000b; F. Haas, MPhil thesis, University of Exeter, 1994). These works described the wing structure concerning the designations, general vein proceedings, folding structures and the stability during the unfolded static state. Not considered were the flapping movements of the wing and their influence on the structure and stability during flight.

We assumed that earwigs belong to the slowly flying insects with very flexible and twisting wings, an extensive distribution of resilin and flexion lines for the wings' shape deformation. We also questioned the stability of the two important mechanisms (mid-wing mechanism and claval flexion line) during flapping flight even under extreme wing shape changes.

With our results, more insights in the wing structure during static and dynamic state were obtained. An extensive distribution of resilin in the veins prevents them from getting damaged through concentrated stress during deformation. Folding lines which also act as flexion lines – lines at which the wing bends up or down during flight – help to create a well formed cambered wing and an adaptation of the claval flexion line during flight helps to inhibit an unwanted folding of the wing.

The tanning patterns found by us match with the observations of Kleinow (1966) and Haas (F. Haas, MPhil thesis, University of Exeter, 1994), who named different sclerotisation grades within the wing of *F. auricularia*. In their work, squama and apical areas were strongly sclerotised, ulnar area sclerotised but membranous and the fan mainly membranous. We observed the very same distribution in both *F. auricularia* and in *L. minor* – supporting the assumption that the degree of tanning is indicative of the sclerotisation in this case (Chapman, 1982; Hepburn and Chandler, 1980).

Solitary veins are often strengthened in the direction in which they need to stay rigid, but flexible to twisting and bending forces in the other direction perpendicular to the main load axis (Newman and Wootton, 1986; Wootton, 1992). In the present case, the longitudinal veins are stabilised and connected with each other through a ring cross vein which prevents them from twisting, allowing for reliable anisotropic properties. The stronger sclerotisation in the distal part aids the veins to withstand strong bending forces during up- and downward strokes and maintains the wing shape during flight. Resilin also supports the bending behaviour of the veins so that no material failure will occur during flight.

Decreasing stiffness from the leading to the trailing edge as well as highly distributed resilin enables a high grade of deformation in the wing without material failure in chordwise direction. Combes and Daniel (2003a) already stressed the importance of stiff material distribution. Here the spanwise or chordwise stiffness was not ascertained by force measurements but with the distribution of stiff material within the wing. The leading edge is hardened from the base to the wing tip; it is the main contributor to spanwise stiffness. Our method did not allow to determine the extent with which the resilin-filled central area contributes to this stiffness. To resolve this question, force measurements along the wing span are necessary, as well as comparative studies of the wings of other insects.

Video analyses showed that the wings deform considerably during flight. Strong deformations strain the material and especially stiff materials, which are important for the stabilisation, are at risk to fail. Related microscopic analyses revealed a high concentration of resilin in the veins (Deiters et al., 2015) as well as various stiffened areas which were distributed according to the deformations. The elastic protein resilin supports the sclerotised and stiff veins to cope with deformation by adding elasticity to stiffness. Areas which are exposed to strong deformations during flight are less sclerotized and therefore less stiff than areas which are formative and important for the stability of the wing for example the leading edge. Hence on the basis of structural studies, conclusions are possible about the usage of these areas during flight and/or folding. Veins in the proximal part of the wing for example show an uneven distribution of stiffness. In this area, the deflexion of the claval flexion line increase or decrease dependent on the wing movements and therefore the veins need more flexibility than the veins in the distal part which are moving as a unit during the flapping cycle.

An even slightly cambered wing can bear higher bending forces and positively support the aerodynamic abilities of the wing compared to a completely flat wing (Vogel, 1967; Wootton, 1992; Sunada et al., 1998). Camber can be supported for example through an active pronation of the leading edge and a bending along a flexion line behind it (Betts, 1986). A skilful assembly of the wing veins can also form a cambered wing during flight and therefore positively support the aerodynamic abilities (Sunada et al., 1998; Anderson, 2012). The earwig wings do also employ these mechanisms and we assume that therefore positive aerodynamic abilities are supported. The squama can be pronated actively; together with the flexion line behind the squama and ulnar area camber is supported. The resilin-filled veins help to maintain this cambered wing during flight and can intensify the camber through a strong passive bending of the veins. Thus optimal lift can be generated (Gorb, 1999; Mountcastle and Combes, 2013). The change of camber intensity is strongly influenced through the V-like leading edge where the outer part (consisting of outer and inner apical area) bends downward or slightly upward from the squama and therefore increases or decreases the camber. Camber helps to raise the maximum ratio of lift to drag in fly wings and increases the maximum lift obtainable (Vogel, 1967).

Wootton (1992) mentioned that insects with extensively twisting wings and a high structural flexibility are often associated with the ability to fly slowly and sometimes to operate at a wide range of speeds with a high degree of manoeuvrability. Flexion lines in the wing enable deformation of the wings to achieve the desired manoeuvrability and optimal lift. Folding lines often act as flexion lines during flight (Wootton, 1992). The dermapteran wing has many folding lines. Two important convex folding lines run through the central area. Together with a concave folding line they form the mid-wing mechanism which is one of the main mechanisms to stabilise the wing (Kleinow, 1966). These important convex folding lines also represent flexion lines at which the wing bends up- and downwards during flight and the squama tilts forward. The wing stability could be seriously affected if the wing folded down at these lines during flight. The flexion line running through the central area, bordering the ulnar area and ending in the fan (green dashed line Fig. 1), especially experiences strong changes of shape during flight. These changes are formed through the up- and downward movement of the distal part. When one of the crucial folding lines in the mid-wing mechanism deforms strongly during flight, the danger of a ‘snap-out’ movement of this mechanism is high. It would initiate an unwanted folding process during flight.

Kleinow (1966) described that the claval flexion line inhibits one of the mentioned flexion lines to gain a stable static unfolded wing. During our flight analysis, we could additionally show that this claval flexion line changes its shape and size. At rest, it mainly inhibits the folding/flexion line directly behind the squama as mentioned by Kleinow (1966). During flapping, a larger area around the claval flexion line is deformed and therefore the second crucial flexion line is inhibited. This is probably important for the unfolded dynamic state of the wing: It ensures that even if the mid-wing mechanism ‘snapped-out’ during flight, the wing cannot fold back directly. Additionally it enables the mechanism to ‘snap-in’ again during the next downstroke as long as the other folding lines stay in place.

These results are established through the analysis of video- and image data. We show that the folding structures influence the three dimensional shape of the wing during flight and the wing deformation abilities. A potentially positive or negative influence of the folding structures during flight regarding e.g. lift production cannot be established with this method. In this case a suitable finite element model is required. Especially the influence of the buckling on a cambered profile for the aerodynamic properties has to be examined with a suitable simulation approach.

Our results show that the observed structures within the wings really do support the folding process and the flight behaviour similarly. The interesting facts in both processes are that the high wing deformations as well as the folding mechanisms are nearly completely controlled passively by stored inner energy and without the need for active actuation (or force production) by muscles. Transferring the observed mechanisms to technical folding procedures might allow for passively controlled, lightweight yet precise mechanics.

## MATERIALS AND METHODS

### Animals

Adults of the common earwig [*Forficula auricularia* (Linnaeus, 1758)] were caught with live-catch traps (C. Huth, PhD thesis, Johannes Gutenberg University of Mainz, 2011) in various regions of North Rhine-Westphalia (Germany) and then reared in a terrarium. Bred individuals of *F. auricularia* were supplied in larval state by the Kölliker group (University of Basel, Switzerland). These were reared to the adult winged state for our analyses. Individuals of the lesser earwig [*Labia minor* (Linnaeus, 1758)] were collected on dung hills, especially horse dung in the region around Bocholt (North Rhine-Westphalia, Germany). With a hand hoe the dung was separated and examined. *L. minor* was found predominantly in warm layers with moderate moistness and only little fungal infestations. The animals were collected with a manual exhauster and transferred to terrain in the laboratory. All animals were kept in polyethylene petri-dishes filled with sand and a layer of dung. The environment inside the dishes needed to be kept moist but not wet. The care and use of the experimental animals complied with the institutional and national animal welfare laws, guidelines, and policies.

### Wing preparation

Only individuals with complete and fully functional folded wings were selected for preparation. All animals were anesthetised in a refrigerator (−20°C) for 10 (*L. minor*) to 15 (*F. auricularia*) minutes. The animals were then placed on a wax surface and following Kleinow (1966) the wing was unfolded directly on a water droplet on an adjacent microscope slide and separated from the body with a scalpel. The surface tension of the water greatly facilitates a non-destructive unfolding process.

### Structural analysis

For structural analysis, the separated wings were first digitised using a reflective-light stereo-microscope (Leica S8 APO) with a camera (Leica EC3) and software (Leica Application Suite EZ). Only wings free from visible damages were used. 17 wings of *F. auricularia* as well as of *L. minor* were then geometrically studied using imaging analysis software [ImageJ, standardpackages, (Schneider et al., 2012)].

### Material properties

Sclerotisation grades in the wing and therefore hardness of the cuticle were determined visually by examine the tanning of the cuticular structures (Chapman, 1982; Anderson, 2012; Hepburn and Chandler, 1980).

The presence of resilin was determined in both species by exploiting its auto-fluorescence properties in the ultra-violet range according to the work of Haas et al. (2000b) on *F. auricularia*. Therefore a fluorescent stereo microscope (Leica M165 FC) and an inserted ultraviolet filter (365/50 nm excitation, 420 nm emission filter) were used. Photographs were taken with a Nikon D90 camera.

### Free-flight analysis

Structural stability and deformations were analysed using three-dimensional high-speed cinematography. Animals were recorded while taking off from a dedicated ramp (following observations of Kleinow, 1966) positioned in a small open box (5.5×5.5×1.5 cm), which was placed in an open glass terrarium (30×20×20 cm). To facilitate finding the ramp, the animals were placed inside the small box. The flight experiments were only performed with *L. minor* as it showed a reliable flight motivation.

The take-offs were recorded with high-speed camera heads (IDT MotionXtra NX3, zoom-lens 18-108 mm) from three directions with 1000 frames per second, a resolution of 1280×1024 px and an exposition time of 201 μs. The arena was illuminated with cold-light LEDs (Spot light 10°, 10 W, 24 V). In general, the highest flying motivation was observed in animals collected on rainy days and directly transferred into the dry and warm flight arena.
